# Cardiovascular Immunotoxicity Associated with Immune Checkpoint Inhibitors in Metastatic Melanoma

**DOI:** 10.3390/cancers15072170

**Published:** 2023-04-06

**Authors:** Jean-Matthieu L’Orphelin, Charles Dollalille, Julia Akroun, Joachim Alexandre, Anne Dompmartin

**Affiliations:** 1 Department of Dermatology, Caen-Normandie University Hospital, 14033 Caen CEDEX, France; 2 Department of Cardiology, Caen-Normandie University Hospital, 14033 Caen CEDEX, France

**Keywords:** melanoma, adverse events, immunotherapy, cardiology, CVAE

## Abstract

**Simple Summary:**

PD-1 inhibitors (nivolumab, pembrolizumab) and anti-CTLA-4 (CD152) (ipilimumab) are widely used in metastatic melanoma, and most immune-related adverse events are known. Recently, several cardiovascular AEs (CVAEs, i.e., myocardial infarction, heart failure, stroke, Takotsubo syndrome, and arrhythmia) have been associated with immune checkpoint inhibitor exposure in post-marketing surveillance studies and represent major issues for patients with melanoma during and after cancer treatment. The aim of this study was to estimate the risk of CVAES associated with immune checkpoint inhibitor exposure for melanoma. Among the cancer population of our systematic review, we performed a safety meta-analysis in a subgroup of melanoma patients.

**Abstract:**

Background: Checkpoint inhibitors, such as PD-1 inhibitors (nivolumab, pembrolizumab) and anti-CTLA-4 (CD152) (ipilimumab), are widely used in metastatic melanoma, and most immune-related adverse events are known. Several cardiovascular AEs (CVAEs) associated with immune checkpoint inhibitor exposure have been reported in post-marketing surveillance studies and represent major issues for patients with melanoma during and after cancer treatment. Data on CVAES induced by immune checkpoint inhibitors in melanoma, especially incidence and risk factors, are lacking. Methods: A systematic review of the literature up to 31 August 2020 was performed in Medline, the Cochrane Central Register of Controlled Trials (CENTRAL), and the ClinicalTrials.gov register according to prespecified selection criteria from inception to 7 April 2020. Statistics were performed on 3289 patients from five randomized clinical trials on melanoma. Results: Patients with melanoma treated with immune checkpoint inhibitors had a significant risk of presenting dyslipidemia (Peto OR: 4.74, 95% CI: 2.16–10.41, *p* < 0.01, I2 = 0%, *p* = 0.94). The Peto OR was numerically significant for pericarditis, myocarditis, heart failure, myocardial infarction, cerebral ischemia, high pulmonary pressure, blood high pressure, arrhythmias, endocarditis, and conduction disturbances, but the confidence interval was not significant. The risk of CVAEs was not statistically different between melanoma treated with immune checkpoint inhibitors and other tumors treated with immune checkpoint inhibitors (range of *p*-value from 0.13 to 0.95). No interaction between follow-up length and CVAE reporting was found. Conclusions: Our study underlines that checkpoint inhibitors used for melanoma increase CVAEs, especially dyslipidemia, which could pave the way to chronic inflammatory processes, atherosclerosis, and, finally, ischemic cardiopathy. These cardiovascular adverse events could be acute or delayed, justifying the monitoring of lipidic biology and a baseline cardiology consultation.

## 1. Introduction

Immune checkpoints inhibitors (ICIs), such as PD-1 inhibitors (e.g., nivolumab or pembrolizumab [1] and ipilimumab (anti-CTLA-4 or CD152)), block prototypical T-cell checkpoints and are now the gold-standard treatment.

Most adverse events (AEs) are immune-related (irAEs) and occur at a median of 40 days [2], whereas 10% are severe (grade III/IV) [3,4].

Overall survival is constantly increasing due to ICI use, and patients with melanoma can become old with a history of treated melanoma; this enables the expression of much later-onset side effects, such as certain cardiovascular effects.

Cardiovascular (CV) irAEs (i.e., myocarditis, pericarditis, and vasculitis) due to ICIs are rare, especially myocarditis (around 1%), but they are usually severe, with high morbidity and mortality rates [5] due to the immune infiltration of CD4+ T cells into the heart [6] when patients are treated with ICIs. Some late-onset and non-inflammatory cardiac irAEs have been reported [7,8].

More recently, several cardiovascular AEs (CVAEs, i.e., myocardial infarction, heart failure, stroke, Takotsubo syndrome, and arrhythmia) have been associated with ICI exposure in post-marketing surveillance studies and represent major issues for patients with cancer during and after cancer treatment. The frequency of CVAEs is higher in cancer patients than in the general population [9,10]. We performed a meta-analysis, which was our previous study, for all types of cancers [11], and we reported a significant risk of myocarditis (Peto OR: 4.42, 95% CI: 1.56–12.50), pericarditis (Peto OR: 2.16, 95% CI: 1.42–3.29), cardiac failure (Peto OR 1.51, 95% CI: 1.01–2.26), and dyslipidemia (Peto OR: 3.68, 95% CI: 1.89–7.19).

Alcoholism and smoking are known risk factors for most solid cancers, altering the epigenome. They are also cardiovascular risk factors. However, these risk factors are not found in melanoma. We conducted this ancillary study to determine whether melanoma treated with immunotherapy presents a similar risk of CVAEs compared with other solid cancers.

## 2. Materials and Methods

### 2.1. Registration

The study protocol was prospectively registered to the International Prospective Register of Systematic Reviews (registration number: CRD42020165672) and was conducted according to the Preferred Reporting Items for Systematic Reviews and Meta-Analyses (PRISMA) Guidelines. No ethics committee approval or subject informed consent was obtained, as this was a retrospective analysis of already published studies.

### 2.2. Data Sources, Search Strategy, and Data Extraction

For the meta-analysis source, a systematic review of the literature up to 31 August 2020 was performed in Medline, the Cochrane Central Register of Controlled Trials (CENTRAL), and the ClinicalTrials.gov register according to prespecified selection criteria from inception to 7 April 2020. We used both controlled terms (i.e., MeSH terms in MEDLINE) and free-text terms related to ICIs, with the language restricted to English.

Second, we focused on terms related to ICIs used in melanoma only (anti-PD-1 antibodies (nivolumab and pembrolizumab) and anti-CTLA-4 antibodies (ipilimumab)) in the title or abstract (or both) that were considered the sole research domain, and the search strategy included the Cochrane Highly Sensitive Search Strategy for identifying RCTs in Medline. RCTs including at least one ICI-containing arm (including ICI monotherapy or a combination of ICIs) that enrolled adult patients (age ≥18 years) with melanoma and provided information on CVAEs were eligible for inclusion. Case reports or case series, case–control studies, observational studies, single-arm studies, and nonrandomized trials were excluded. Patients with risk factors related to the development of cardiovascular disease prior to the initiation of ICIs were not excluded from the analysis.

First, all available CVAEs, classified according to the Common Terminology Criteria for Adverse Events (CTCAE), in RCTs on ICIs reported on ClinicalTrials.gov were extracted. Second, if the reported CVAEs were not available on ClinicalTrials.gov, all reported CVAEs were extracted from published RCTs. RCTs without data related to the CVAEs of interest were not included.

Additional data from eligible studies were collected, including the ICI regimen, control arm regimen, median age, median/mean follow-up duration, and overall number of patients analyzed. All results, including follow-up data posted on ClinicalTrials.gov, were collected at the time of the searches.

Two authors evaluated the risk of bias in individual studies using the Pharmacoepidemiological Research on Outcomes of Therapeutics by a European Consortium (also known as PROTECT) checklist tool, which is specially designed to assess bias in safety meta-analyses [12]. In cases of disagreements, a third author was consulted.

**Figure 1:**
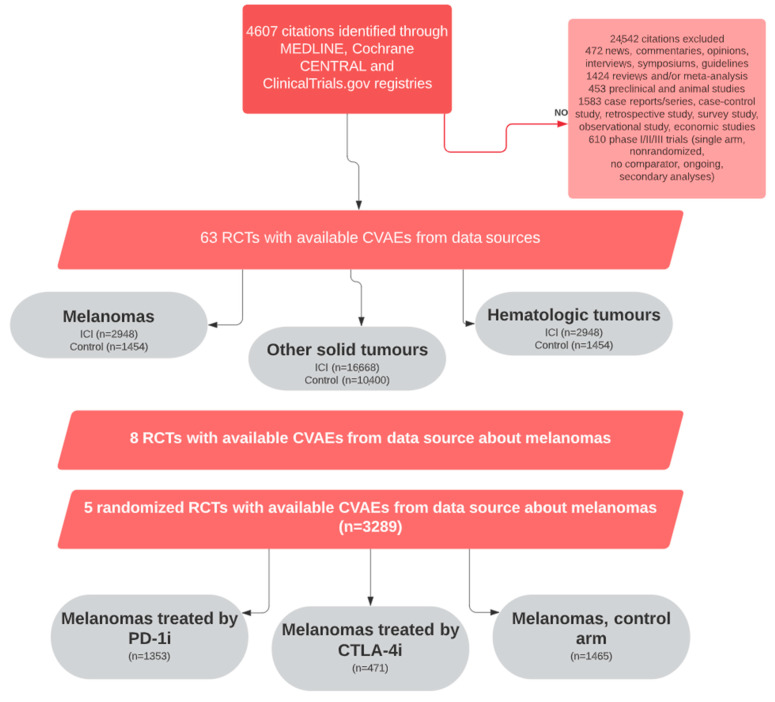
Study flowchart: PRISMA flow diagram of systematic review and meta-analysis in ClinicalTrials.gov registries, Medline, and Cochrane CENTRAL up to 7 April 2020.

The study flowchart is given in Figure 1.

### 2.3. Outcomes

The primary outcome was the summarized risk of CVAEs associated with ICI exposure (ICI monotherapy—pembrolizumab, nivolumab, or ipilimumab—or a combination of ICIs). The control arms could be either placebo or non-placebo agents (kinase inhibitors, vascular endothelial growth factor pathway inhibitors, or chemotherapy).

The CVAEs we gathered were ventricular and supraventricular arrhythmias, cardiogenic shock, dyslipidemia, venous thrombo-embolic issues, high blood pressure, high pulmonary pressure, myocardial infarction, cerebral ischemia, heart failure, myocarditis, pericarditis, QT/QTc prolongation and torsade de pointe, valvopathy, and conduction disturbances. All CVAEs were defined by MedDRA terminology.

### 2.4. Statistical Analysis

We performed a random-effects meta-analysis to compute the Peto odds ratios (ORs) with 95% confidence intervals (CIs), which has been described as the most accurate method for binary studies on rare events (<1%) [13]. Assuming that CV irAEs were not frequent events (incidence <10%), we interpreted the OR as a measure of risk [14,15].

In this study, specific subgroup analyses were performed regarding the types of tumors: MM vs. other tumors. “Other tumors” comprised bronchial adenocarcinomas, small cells carcinomas, colorectal cancer, gastric cancer, glioblastoma, hepatocellular carcinoma, mesothelioma, myeloma, ovarian cancer, pancreatic adenocarcinoma, prostate cancer, breast cancer, and urothelial cancer.

A two-sided *p*-value of <0.05 in Z-tests (for overall effect) or v2 tests (for overall subgroup comparison) in all analyses was considered statistically significant.

## 3. Results

### 3.1. Descriptions of Included Studies

The PRISMA flow diagram of study selection is presented in Figure 1. The details of the study characteristics are presented in Table 1. Among the 63 RCTs included in our previous study, 8 phase III studies dedicated to melanoma were kept. Three studies were excluded since no control arm was available; only five studies with a control arm out of the eight clinical trials were considered. A total of 3289 adult patients with melanoma were enrolled, with 1824 (55%) patients in the ICI arm, of whom 1353 were treated with PD-1i and 471 were treated with CTLA-4i.

In the control arm, 1465 patients received placebo or chemotherapy; 979 patients (67%) received placebo, and 486 (33%) received chemotherapy. In the ICI arm, 28% of the immunotherapy regimens were nivolumab, 29% were pembrolizumab, 28% were ipilimumab monotherapy, and 15% were double immunotherapy with ipilimumab–nivolumab.

The population was 62% male, and the mean age was 58.9 years old. Follow-up ranged from 5.2 months to 32.8 months.

### 3.2. Risk of CVAEs Associated with ICI Exposure and Incidence of CV irAEs with an Increased Risk Associated with ICI Exposure in Melanoma

Patients with MM treated with ICI had a significant risk of presenting dyslipidemia (Peto OR: 4.74, 95% CI: 2.16–10.41, *p* < 0.01, I2 = 0%, *p* = 0.94). The Peto OR was numerically significant for pericarditis, myocarditis, heart failure, myocardial infarction, cerebral ischemia, high pulmonary pressure, high blood pressure, arrhythmias, endocarditis, and conduction disturbances, but the confidence interval was not significant.

The risk of CVAEs was not statistically different between MM treated with ICI and other tumors treated with ICI (range of *p*-value from 0.13–0.95). No interaction between the follow-up length and CVAE reporting was found.

When data were disaggregated by sex, no statistical differences were found for cardiac conductive disorders (*p* = 0.16), cardiac death or shock (*p* = 0.62), cardiac supra-ventricular (*p* = 0.19) or ventricular (*p* = 0.59) arrhythmias, heart failure (*p* = 0.3), hypertension (*p* = 0.52), myocarditis (*p* = 0.84), or cerebral arterial ischemia (*p* = 0.57), and data were not available for dyslipidemia.

In three studies, patients had previous treatment before ICI, but it was not statistically significant.

**Figure 2:**
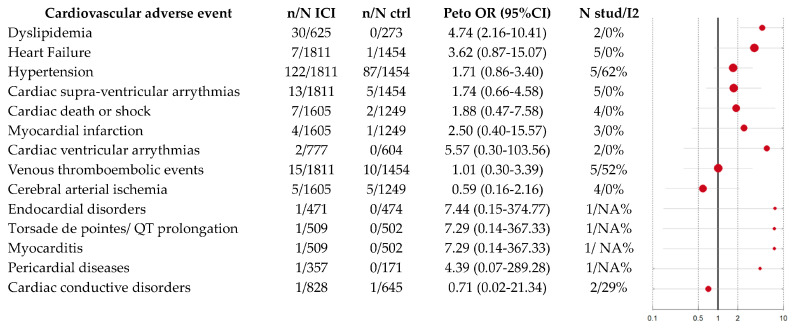
Risk of CVAEs for MM treated with ICI. The line represents the confidence interval and the size of the circle/dot represents the strength of the association (i.e., peto OR).

All results are summarized in Figure 2, and details are given in Table 2. We reported no statistical differences between melanoma and “other solid cancers” regarding CVAEs (*p* ranging from 0.13 to 0.95).

## 4. Discussion

There was a higher incidence of melanoma in males than in females, with differences between the location of primary tumors and mortality, due to discovery at later stages in males. However, the purpose of our study was to investigate the cardiovascular risk factors of immunotherapy in melanoma and assess the potential difference from other cancers independent of overall survival and mortality. Our study showed that the population was comparable between patients with melanoma and patients with other cancers When treated with immunotherapy, melanoma patients had the same risk of developing CVAEs as those with other solid cancers, and alcohol and tobacco intake played a role in both cardiovascular risk factors and oncogenesis.

Dyslipidemia was significantly associated with ICIs, whereas the previous study showed a significant Peto OR for a higher risk of myocarditis, pericarditis, heart failure, dyslipidemia, and myocardial infarction. Autoimmunity can be ascribed among the potential mechanisms of ICI-associated CVAEs, and to a greater extent, although not unique, myocarditis [16]. Nevertheless, we have to point out that several CVAEs (arrhythmias, myocarditis, coronary artery disease, valvopathies, and cardiomyopathy) have a sharply different pathologic basis, making it difficult to define a common origin when they are considered secondary events associated with the use of anti-neoplastic drugs.

In our cohort, the sex ratio was 2/3, whereas the sex ratio of melanoma is around 1. It is well-known that males over 45 years old have a higher cardiovascular risk regarding SCORE (European Society of Cardiology 2019) [17], which could maximize the baseline cardiovascular risk. Women may have more misleading presentations of cardiovascular pathology, leading to delayed management or misdiagnosis. In our study, we did not find a gender-related excess risk. Nevertheless, atypical presentations in females might not have been taken into account in clinical studies and should be a cause for vigilance in future studies.

We reported that 35% of melanomas were treated with ipilimumab monotherapy, which is not a common or universal practice. We could suppose that this over-representation of ipilimumab overestimates CVAEs in real-life data since ipilimumab is more of a provider of irAEs than PD-1i.

We reported a strong tendency toward myocardial infarction and stroke, whereas the previous study reported significant results. The non-significance can be explained by a lack of power (fewer patients were included when focusing on melanoma only). Atherosclerosis is reported in 45 to 75% of patients with cancers [18], and the incidence rate of atherosclerosis with ICIs is underestimated because of the slow development and potential manifestations after the end point of clinical trials [19]. Classically, inflammation in atherosclerosis is mediated by macrophages present in atheroma plaque [20], but when treated with ICIs, T lymphocytes stimulated by ICIs are involved in the atherosclerosis process [21], and atheroma plaque is mostly made of lymphocytes [22]. CVAEs increased threefold under ICI treatment, and atheroma plaque progression was three times higher with ICIs [23].

Atherosclerosis phenomena under ICI treatment are increased by dyslipidemia [24]. We reported that the risk of development of dyslipidemia was four times higher in melanoma treated with ICIs. In [25], an animal model carrying a myeloid cell-specific PD-1 ablation implicated in the anti-tumor immune response by effector myeloid cells underlined that PD-1-deficient mice synthesized more cholesterol by glycolysis mechanism alteration. It has been suggested that baseline dyslipidemia under ICIs is a good prognosis factor [26], as most irAEs are [27]. In our study, we reported the occurrence of dyslipidemia under treatment, and we did not study this parameter as a baseline characteristic.

Statins are known to have a benefit on CV mortality [28] thanks to anti-inflammatory effects and hypolipemiant properties, stifling the increase in atherosclerosis [29]. Some studies have suggested that statins could have a protective effect against MM [30,31] by modulating the immune response with a higher innate response and tumoral immunity. Nevertheless, the use of cholesterol-lowering drugs, such as statins or fibrates, in melanoma is controversial [32,33], specifically in terms of its potential to increase PCSK9 [34], which is associated with platelet reactivity, leading to acute coronary syndrome. Novel lipid-lowering drugs, such as PCSK9 inhibitors, could be proposed, as Quagliariello [35] did, putting forward the argument that PCSK9 inhibition in patients with cancer treated with ICI therapies enables a reduction in atherosclerotic cardiovascular events and potentially improves ICs-related anticancer functions. This meta-analysis did not have the sensitivity to determine whether subjects who developed dyslipidemia under immunotherapy were carriers of a predisposing genetic mutation [36].

Dyslipidemia and atherosclerosis risks are not currently monitored during ICI treatment. Regarding our results, it seems relevant to propose quarterly lipid biology, ECG monitoring, and cardiac echography at baseline, before treatment initiation. Troponin could be also monitored since 94% of myocarditis patients treated with ICIs have increased troponin [37] and normal morphologic parameters before event occurrence. Efficient cooperation between dermatologists and cardiologists should be implemented. Further prospective studies should be implemented to assess whether any CVAEs exist before ICI treatment initiation; cholesterol-lowering drugs have to be proposed, considering that ICI treatment is a cardiovascular risk factor in its own right.

We can consider ICIs as a large part of the CV risk factor since the risk of CVAEs is similar between melanoma patients and those with other solid cancers, i.e., a population in which alcohol and tobacco consumption, a major cardiovascular risk factor, is less represented than in other solid cancers. Currently, there is no standardized consensus on the choice of surveillance strategies and management algorithms for CVAEs in patients participating in oncological RCTs. Annual cardio-oncology monitoring is recommended. Further studies could be implemented to study late-onset CVAEs [38] since irAEs can occur 2 years after the initiation of immunotherapy, even if it has been stopped, suggesting that risk factors are sustainable over time.

## 5. Conclusions

The incidence CVAEs ranged from 3 to 20 per 1000 patients, but it might be underestimated in clinical trials, as cardiac monitoring is usually lacking in melanoma trials involving ICIs. We report a significant risk of dyslipidemia, and our study suggests that we could consider ICIs to be a large part of the cardiovascular risk factor. Dyslipidemia and, more broadly, CVAE could occur in melanoma as in other cancers treated with ICIs. This suggests that the risk of dyslipidemia with ICIs cannot be attributed to alcoholism and smoking since they are not risk factors for melanoma.

Our study underlines that ICIs used in melanoma increase CVAEs, especially dyslipidemia, which could pave the way to chronic inflammatory processes, atherosclerosis, and, finally, ischemic cardiopathy. This assertion is all the more interesting because the average lifespan of melanoma patients is increasing, allowing slowly evolving side effects, such as dyslipidemia, to appear.

These cardiovascular irAEs could be acute or delayed, justifying the monitoring of lipid biology and a baseline cardiology consultation. Further prospective studies could assess cholesterol-lowering drug use to reduce late-onset CVAEs in melanoma patients treated with immunotherapy.

## Figures and Tables

**Table 1:** Characteristics of the included randomized clinical trials.

ClinicalTrials.gov Identifier	Study	Study Design	Comparison	Advanced or Metastatic Cancer	Prior Systemic Therapy (%)	Mean Patient Age (years)	Follow up Duration (months)	Number of Patients in the ICI Mono Therapy Group	Number of Patients in the Combination ICI Therapy Group	Number of Patients in the Control Group
NCT00636168	*Eggermont Lancet Oncol*. *2015*	Phase 3 RCT	Ipilimumab 10 mg/kg vs. placebo	No	0	51.1	32.8	471	-	474
NCT01704287	*Ribas Lancet Oncol. 2015*	Phase 2 RCT	Pembrolizumab 2 or 10 mg/kg vs. chemotherapy (paclitaxel + carboplatin, paclitaxel, carboplatin, dacarbazine or temozolomide)	Yes	46–50	60.1	10	357	-	171
NCT01721746	*Weber Lancet Oncol. 2015*	Phase 3 RCT	Nivolumab 3 mg/kg vs. chemotherapy (dacarbazine, or carboplatin + paclitaxel)	Yes	100	59.2	8.4	268	-	102
NCT01721772	*Robert N. Engl. J. Med. 2015*	Phase 3 RCT	Nivolumab 3 mg/kg + placebo vs. dacarbazine + placebo	Yes	16.8	62.7	5.2	206	-	205
NCT01844505	*Larkin N. Engl. J Med. 2015*	Phase 3 RCT	Nivolumab 3 mg/kg vs. Nivolumab 1 mg/kg + Ipilimumab 3 mg/kg vs. Ipilimumab 3 mg/kg	Yes	0	59.6	12.2	624	313	-
NCT01927419	*Postow N. Engl. J. Med. 2015*	Phase 3 RCT	Ipilimumab 3 mg/kg + placebo vs. nivolumab 1 mg/kg + Ipilimumab 3 mg/kg	Yes	0	63.7	11	46	94	-
NCT02362594	*Eggermont N. Engl. J. Med. 2018*	Phase 3 RCT	Pembrolizumab 200 mg vs. placebo	No	0	53.8	15	509	-	502
NCT02374242	*Long Lancet Oncol. 2018*	Phase 2 RCT	Nivolumab 3 mg/kg vs. Nivolumab 1 mg/kg + Ipilimumab 3 mg/kg	Yes	-	61.1	14	25	35	

**Table 2:** Comparison of CVAEs for MM patients versus patients with other cancers.

	Immune Checkpoint Inhibitors Arm (n/N)		Control Arm (n/N)		n Study/.I2		OR (IC95%)		*p*
	*Melanomas*	*Other Tumors*	*Melanomas*	*Other Tumors*	*Melanomas*	*Other Tumors*	*Melanomas*	*Other Tumors*	
Supraventricular arrhythmias	13/1811	85/11,217	5/1454	60/7406	5/0%	29/27%	1.74 (0.66–4.58)	0.77 (0.50–1.20)	0.13
Ventricular arrhythmias	2/777	4/2591	0/604	2/1572	2/0%	6/31%	5.57 (0.30–103.56)	1.18 (0.16–8.80)	0.39
Cardiogenic shock	7/1605	60/10,338	2/1249	27/6873	4/0%	26/0%	1.88 (0.47–7.58)	1.45 (0.93–2.24)	0.73
Dyslipidemia	30/625	9/1422	0/273	2/661	2/0%	2/0%	4.74 (2.16–10.41)	1.91 (0.54–6.79)	0.23
Venous thromboembolic events	15/1811	231/12,154	10/1454	162/8303	5/52%	34/26%	1.01 (0.30–3.39)	0.97 (0.74–1.25)	0.95
High blood pressure	122/1811	376/7668	87/1454	208/4758	5/62%	18/76%	1.71 (0.86–3.40)	1.12 (0.74–1.70)	0.30
High pulmonary pressure		2/2710		5/1696		6/3%		0.26 (0.05–1.23)	0.42
Myocardial infarction	4/1605	66/11,093	1/1249	30/7631	3/0%	28/0%	2.50 (0.40–15.57)	1.47 (0.97–2.22)	0.58
Cerebral ischemia	5/1605	93/10,731	5/1249	36/7454	4/0%	29/0%	0.59 (0.16–2.16)	1.68 (1.17–2.40)	0.13
Heart failure	7/1811	82/9903	1/1454	28/6634	5/0%	27/0%	3.62 (0.87–15.07)	1.90 (1.28–2.80)	0.39
Myocarditis	1/509	13/4857	0/502	1/3587	1/NA%	11/0%	7.29 (0.14–367.33)	4.25 (1.44–12.51)	0.79
Pericarditis	1/357	70/8906	0/171	22/6702	1/NA%	23/2%	4.39 (0.07–289.28)	2.17 (1.41–3.32)	0.74
Torsades de pointes/QT prolongation	1/509	2/1211	0/502	1/589	1/NA%	3/37%	7.29 (0.14–367.33)	0.96 (0.05–19.87)	0.42
Conduction disturbance	1/828	2/3004	1/645	4/1782	2/29%	6/0%	0.71 (0.02–21.34)	0.29 (0.05–1.52)	0.64
Valvulopathy		1/1769		2/1363		3/41%		0.37 (0.02–7.64)	0.39

## Data Availability

The data presented in this study are available upon request from the corresponding author. Public data are available on ClinicalTrials.gov.
